# Can randomized trials realistically detect patient benefit from robotic-assisted reverse shoulder arthroplasty?

**DOI:** 10.1016/j.jsea.2026.100106

**Published:** 2026-09-30

**Authors:** Eoghan T. Hurley, Matthew Frederickson, Joaquin Sanchez-Sotelo, George S. Athwal, Mariano E. Menendez

**Affiliations:** a Department of Orthopaedic Surgery, Duke University, Durham, NC, USA; b Department of Orthopaedic Surgery, University of California Davis, Sacramento, CA, USA; c Department of Orthopedic Surgery, Mayo Clinic, Rochester, MN, USA; d Roth / McFarlane Hand and Upper Limb Centre, St Joseph's Health Care, London, ON, Canada

**Keywords:** Shoulder arthroplasty, Robot, Randomized trial, Revision, ASES, Plan, Protocol

## Abstract

**Background:**

Robotic-assisted reverse total shoulder arthroplasty (rTSA) aims to improve implant positioning and plan fidelity, but whether these technical advantages translate into measurable patient benefit remains unknown. The purpose of this study was to determine how large randomized trials comparing robotic-assisted with conventional rTSA would need to be to detect differences in revision rates and patient-reported outcomes.

**Methods:**

A 2-arm parallel randomized trial was modeled with 1:1 allocation, 2-sided alpha of 0.05, and 80% power. Revision endpoint calculations used standard 2-proportion formulas under a baseline revision rate of 4%, with hypothetical absolute risk reductions (ARR) attributable to robotic assistance ranging from 0.5% to 3.0%. American Shoulder and Elbow Surgeons endpoint calculations used standard 2-mean formulas, modeling robotic-attributable between-group differences of 1 to 5 points under standard deviation (SD) assumptions of 15 and 20 points.

**Results:**

Under a baseline revision rate of 4%, detection of a 0.5% ARR in revision surgery required 22,663 patients per group; a 1.0% ARR required 5,301; a 2.0% ARR required 1,141; and a 3.0% ARR required 424. For American Shoulder and Elbow Surgeons with SD 15, detection of a 1-point between-group difference required 3,532 patients per group; a 2-point difference required 883; a 3-point difference required 393; a 4-point difference required 221; and a 5-point difference required 142. Under SD 20, corresponding values ranged from 6,280 to 252 patients per group.

**Conclusion:**

Randomized trials of robotic-assisted rTSA would require very large patient enrollment to detect the small reductions in revision surgery and modest improvements in patient-reported outcomes most plausibly attributable to the technology. These findings suggest that a feasible conventional trial may be poorly suited to detect, or confidently exclude, small advantages of robotic assistance. The next phase of study should therefore prioritize longer-term comparative analyses and focused evaluation of anatomically or clinically higher-risk subgroups, in whom the technology's effect may be larger and more detectable.

Reverse total shoulder arthroplasty (rTSA) has become the most commonly performed shoulder replacement in the United States.^11^, ^19^, ^22^ Despite favorable aggregate outcomes, implant longevity and functional recovery are influenced at least in part by component positioning. Biomechanical studies and clinical series have found associations of variability in glenoid baseplate inclination and version, glenosphere lateralization, and humeral inclination with complications such as notching, instability, acromial stress fractures, and aseptic loosening.^5^, ^10^, ^15^, ^17^ Revision rTSA is technically demanding and costly, and registry data indicate that component malposition is present in the majority of implants requiring revision surgery, with 1 recent series reporting glenoid baseplate malposition in 93% and humeral component malposition in 62% of revision cases.^16^

Robotic-assisted rTSA has recently been incorporated into the surgical practice of some, with the rationale for this technology framed around 3 domains: precision, personalization, and reproducibility.^13^ Precision is critical because even minor deviations in baseplate placement influence fixation, range of motion, and instability risk; superior baseplate inclination has been specifically associated with post-operative instability, which accounts for approximately 30% of all rTSA revisions.^17^, ^20^ Personalization matters, given the expanding complexity of rTSA indications, including cases with severe glenoid bone loss and deformity where a one-size-fits-all technique is insufficient. Reproducibility is particularly relevant for lower-volume surgeons, who perform the majority of rTSAs in the United States. The average annual case volume of rTSA surgeons has been reported at less than 10 procedures per surgeon, and among whom outcomes and complication rates are demonstrably inferior to those of high-volume centers.^6^, ^8^, ^9^ Early positioning data confirm that robotic assistance improves implant accuracy relative to conventional technique and patient-specific instrumentation, and a recent single-surgeon series demonstrated that operative time achieves neutrality relative to a conventional benchmark after approximately 20 to 25 cases.^1^, ^4^ However, whether these technical gains translate into measurable improvements in patient-reported outcomes or revision rates remains unknown.^18^

A major discussion point regarding robotic-assisted rTSA is not whether it can improve implant positioning, but whether such improvement is substantial enough to produce a detectable difference in outcomes that matter to patients.^1^ No prior analysis has examined the trial sizes required to detect clinically relevant differences in the outcomes most commonly proposed for randomized evaluation of robotic-assisted rTSA. The purpose of this study was to determine how large randomized trials comparing robotic-assisted with conventional rTSA would need to be to detect differences in revision rates and patient-reported outcomes. The hypothesis was that revision-based trials would require prohibitively large group sizes and that even American Shoulder and Elbow Surgeons (ASES) score-based trials would demand substantial enrollment when the expected robotic-attributable benefit is small.

## Methods

### Study design

A theoretical sample size analysis was performed for a hypothetical 2-arm parallel randomized trial comparing robotic-assisted rTSA with conventional rTSA. All calculations assumed 1:1 allocation, a 2-sided alpha of 0.05, and 80% statistical power. The objective was not to estimate the true treatment effect of robotic assistance but to examine how required enrollment changes across a range of plausible effect sizes and endpoint assumptions.

### Revision endpoint

Required sample size per group for revision surgery was calculated using standard formulas for comparison of 2 independent proportions. A baseline revision rate of 4% was selected for conventional rTSA, consistent with published intermediate-term registry data. Hypothetical absolute risk reductions (ARRs) attributable to robotic assistance were modeled at 0.5%, 1.0%, 1.5%, 2.0%, and 3.0%, reflecting the assumption that any genuine benefit would most likely be incremental rather than a drastic change in ARRs.

### ASES score endpoint

Required sample sizes per group for post-operative ASES scores were calculated using standard formulas for comparison of 2 independent means. Because both robotic-assisted and conventional rTSA are expected to produce substantial symptomatic improvement, the incremental between-group benefit specifically attributable to robotic assistance would likely be considerably smaller than published Minimal Clinically Important Difference thresholds for ASES in shoulder arthroplasty (range, 11–21 points).^21^ Robotic-attributable between-group differences were therefore modeled at 1, 2, 3, 4, and 5 points. Calculations were repeated under standard deviation (SD) assumptions of 15 and 20 points to capture plausible variability in post-operative scores.

### Statistical analysis

All sample size calculations were performed using standard analytic formulas for 2-group comparisons under equal allocation using GraphPad Prism version 11.0.0 for Windows, GraphPad Software, Boston, Massachusetts, USA, www.graphpad.com. For the revision endpoint, required sample size per arm was derived from the difference between 2 proportions. For the ASES score endpoint, required sample size per arm was derived from the difference between 2 means. Results are presented as the number of patients that would be required per study arm for each modeled scenario.

## Results

### Revision endpoint

Required sample size per group increased steeply as the assumed ARR diminished. Under a baseline revision rate of 4%, detection of a 3.0% ARR would require 424 patients per group. A 2.0% reduction would require 1,141 per group, a 1.5% reduction would require 2,193, a 1.0% reduction would require 5,301, and a 0.5% reduction would require 22,663 patients per group. Results are presented in Table I.

**Table I:** Required sample size for revision as the primary endpoint

Endpoint	Assumption	Required sample size per arm
Revision	ARR 3.0%	424
Revision	ARR 2.0%	1,141
Revision	ARR 1.5%	2,193
Revision	ARR 1.0%	5,301
Revision	ARR 0.5%	22,663

*ARR*, absolute risk reduction.

### ASES score endpoint

Required sample size per group for ASES also rose substantially as the modeled between-group difference narrowed. Under an SD of 15 points, detection of a 5-point difference would require 142 patients per group; a 4-point difference would require 221; a 3-point difference would require 393; a 2-point difference would require 883; and a 1-point difference would require 3,532 per group. Under an SD of 20 points, corresponding group sizes were 252, 393, 698, 1,570, and 6,280. Compared with revision-based trials, ASES score-based trials were more feasible across most scenarios, but required enrollment numbers remained substantial when the expected robotic-attributable difference was 2 points or less (Table II).

**Table II:** Required sample size for ASES as the primary endpoint

Endpoint	Assumption	Required sample size per arm
ASES (SD 15)	Difference 5 pts	142
ASES (SD 15)	Difference 4 pts	221
ASES (SD 15)	Difference 3 pts	393
ASES (SD 15)	Difference 2 pts	883
ASES (SD 15)	Difference 1 pt	3,532
ASES (SD 20)	Difference 5 pts	252
ASES (SD 20)	Difference 4 pts	393
ASES (SD 20)	Difference 3 pts	698
ASES (SD 20)	Difference 2 pts	1,570
ASES (SD 20)	Difference 1 pt	6,280

*ASES*, American Shoulder and Elbow Surgeons; *SD*, standard deviation; pts, patients.

## Discussion

The most important finding of this analysis is that randomized evaluation of robotic-assisted rTSA would likely require very large enrollment numbers to detect the magnitudes of patient benefit most plausibly attributable to this technology. Under realistic assumptions, revision-based trials are effectively difficult to complete without multicenter infrastructure and extended follow-up, and even ASES score-based trials would become demanding when the anticipated incremental gain is small. These observations are relevant, as discussions of this technology frequently shift from enthusiasm to calls for randomized evidence without sufficient consideration of feasibility.

A major point of discussion in this field is whether the most common causes of rTSA failure are amenable to improvement through more precise component positioning. Instability accounts for approximately 30% of all revision rTSA, infection for a substantial further proportion, and humeral complications for much of the remainder.^20^ Critics of robotic technology have argued that these predominant failure modes are driven by patient selection, biological factors, soft tissue management, and fixation technique, not by millimeter- or degree-level imprecision that robotics are designed to address.^12^, ^14^ Glenoid loosening, in which positioning most plausibly plays a role, accounts for fewer than 15% of rTSA failures, and loosening occurs despite acceptable component position in many cases.^3^ Proponents of robotic-assisted technology suggest that analyzing only revision rates underestimates the contribution of malposition, noting that component malposition is present in the vast majority of implants requiring revision and that the complication and outcome literature is largely authored by high-volume experts whose results are unlikely to reflect the true community-level burden of malposition-related failure.^12^, ^14^, ^16^

The magnitude of expected benefit from robotic assistance is unlikely to be uniform across surgeons, centers, and patients, and a small average effect across an unselected population is not evidence that the effect is trivial for everyone within it. The mean annual rTSA surgeon volume in the United States is approximately 6 cases, and surgeons performing fewer than roughly 29 rTSA cases carry significantly higher adjusted odds of 90-day readmission for infection and dislocation, higher transfusion rates, and higher 90-day costs than their higher-volume peers. In a separate nationwide analysis, lower-volume surgeons operated on patients with greater baseline comorbidity and had higher adjusted reoperation rates at 90 days and 1 year, along with higher rates of prosthetic joint infection and periprosthetic fracture.^6^, ^8^, ^9^ As instability and infection together account for a large share of rTSA revisions, and because component malposition is present in the majority of implants requiring revision, a technology that improves positioning consistency would be expected to yield its largest absolute benefit precisely where baseline complication rates are highest: among low-volume surgeons and low-volume centers.^6^, ^8^, ^9^ If robotic assistance narrows the gap between low- and high-volume performance, the effect on population-level revision rates could be meaningful even where the average per-case gain measured in a mixed-volume trial appears small.

The design of our ASES score analysis around small robotic-attributable differences rather than published minimal clinically important difference (MCID) values merits further discussion. MCID thresholds describe meaningful within-patient improvement and are not equivalent to the between-group effect one would expect when comparing 2 effective interventions.^2^, ^7^ Both arms of a randomized trial comparing robotic-assisted and conventional rTSA would be expected to improve outcomes substantially; the question when designing these trials is whether the incremental gain from robotic assistance is large enough to be detected with feasible enrollment. Assuming between-group differences equivalent to published MCID values would inflate the perceived effect and lead to underpowered trials.

Overall, these findings suggest several considerations for future research. Revision as a primary endpoint may be ill-advised for early comparative studies, considering current baseline event rates and usual follow-up durations. Patient-reported outcomes are more statistically efficient, but investigators should select realistic effect sizes and report sample size assumptions transparently. An alternative pathway that sidesteps this detectability problem is to focus current research on intermediate endpoints more directly linked to the proposed mechanism of benefit (component positioning accuracy, plan fidelity, technical outlier rates, and instability-related metrics), for which robotic assistance has already demonstrated improvement.^1^ Prospective multicenter registries capturing both technical metrics and clinical outcomes, linked to resource utilization data, represent the most tractable route to cost-effectiveness evidence in the near term. As the robotic shoulder literature matures, appropriately powered trials using realistic effect sizes will ultimately be required to establish whether precision gains translate into patient benefit at a scale that justifies the cost of this technology.

### Limitations

This analysis has several limitations. The calculations were entirely theoretical and did not estimate actual treatment effects of robotic assistance, which remain unknown. The baseline revision rate and ASES score variability assumptions were illustrative; alternative inputs would yield different numerical results. Revision surgery was modeled as a fixed binary outcome rather than a time-to-event endpoint, and ASES scores were modeled as a single post-operative value without accounting for longitudinal measurement or correlation structures. These simplifications were intentional and preserve the accessibility of the framework, but more nuanced designs, including survival analysis or mixed-effects models, that result in different estimates. The analysis also assumed equal allocation; adaptive designs or unequal randomization would change the absolute enrollment figures without altering the central message.

## Conclusion

Randomized trials of robotic-assisted rTSA would require very large patient enrollment to detect the small reductions in revision surgery and modest improvements in patient-reported outcomes most plausibly attributable to the technology. These findings suggest that a feasible conventional trial may be poorly suited to detect, or confidently exclude, small advantages of robotic assistance. The next phase of study should therefore prioritize longer-term comparative analyses and focused evaluation of anatomically or clinically higher-risk subgroups, in whom the technology's effect may be larger and more detectable.

## Disclaimers:

Funding: No funding was disclosed by the authors.

Conflicts of interest: Joaquin Sanchez-Sotelo has received stock from Precision OS; stock from Orthobullets; and other professional activities and other intellectual property compensation from Exactech, Inc., Stryker, and ACUMED LLC; and stock from Parvizi Surgical Innovation, for work related to the subject of this article.

George S. Athwal has received IP royalties from CONMED Linvatec; IP royalties from Exactech, Inc.; stock or stock options from OTS; stock or stock options from Parvizi Surgical Innovation; stock or stock options from Reach Orthopedics; publishing royalties and financial or material support from Springer; and IP royalties, paid consultant fees, and research support from Stryker, for work related to the subject of this article.

Mariano E. Menendez is a paid consultant for Stryker.

Any additional authors, their immediate families, and any research foundations with which they are affiliated have not received any financial payments or other benefits from any commercial entity related to the subject of this article.
